# Function, Regulation and Biological Roles of PI3Kγ Variants

**DOI:** 10.3390/biom9090427

**Published:** 2019-08-30

**Authors:** Bernd Nürnberg, Sandra Beer-Hammer

**Affiliations:** 1 Department of Pharmacology & Experimental Therapy, Institute of Experimental and Clinical Pharmacology and Toxicology, University of Tübingen, Wilhelmstrasse 56, 72074 Tübingen, Germany; 2 Department of Toxicology, Institute of Experimental and Clinical Pharmacology and Toxicology, University of Tübingen, Wilhelmstrasse 56, 72074 Tübingen, Germany

**Keywords:** PI3K (phosphatidylinositide 3-kinase), class I PI3-kinases, p110γ, p87 (p84), G-proteins, Gβγ, Ras

## Abstract

Phosphatidylinositide 3-kinase (PI3K) γ is the only class IB PI3K member playing significant roles in the G-protein-dependent regulation of cell signaling in health and disease. Originally found in the immune system, increasing evidence suggest a wide array of functions in the whole organism. PI3Kγ occur as two different heterodimeric variants: PI3Kγ (p87) and PI3Kγ (p101), which share the same p110γ catalytic subunit but differ in their associated non-catalytic subunit. Here we concentrate on specific PI3Kγ features including its regulation and biological functions. In particular, the roles of its non-catalytic subunits serving as the main regulators determining specificity of class IB PI3Kγ enzymes are highlighted.

## 1. Introduction

**Figure 1:**
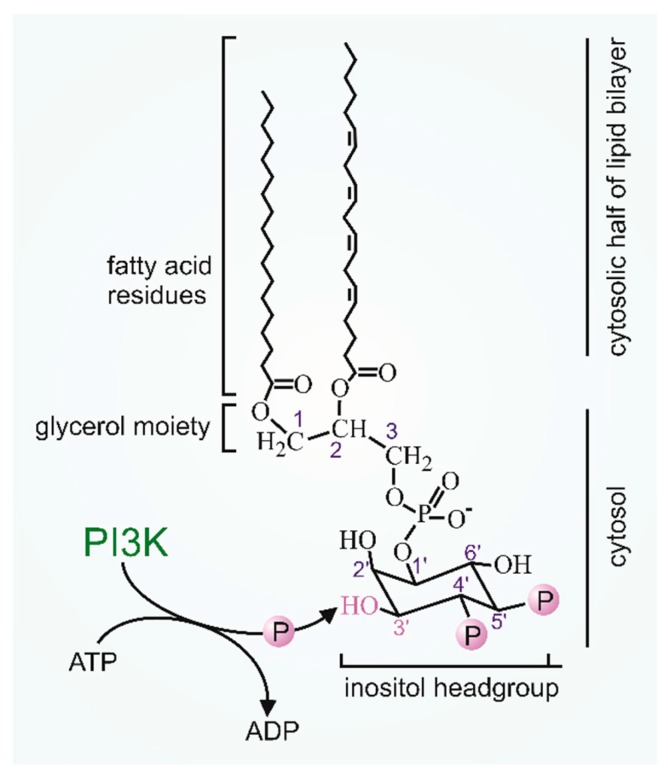
Schematic representation of the PI3K lipid kinase activity. Activation of membrane-associated class I PI3Ks results in phosphorylation of the 3-hydroxyl position of inositol ring in Ptd-4,5-P_2_ generating the essential second messenger of the plasma membrane, Ptd-3,4,5-P_3_.

Lipid kinases, which specifically phosphorylate the inositol moiety of phospholipids at the 3′ position, are assigned to the superfamily of Phosphatidylinositide 3-kinases (PI3K). They play profound roles in the physiological control of body homeostasis but are also targeted for drug therapy for a broad range of human diseases [1,2,3]. PI3K enzymes are subdivided into three classes based on homology, substrate specificity and functional features. The class II and class III members and their roles as regulators of membrane traffic during endocytosis, endosomal recycling and autophagy were recently reviewed in detail [1]. The class I enzymes are controlled by cell surface receptors and comprise four different catalytic isoforms, i.e., p110α,β,γ,δ, and they utilize phosphatidylinositol (4,5)P_2_ (PIP_2_) to produce phosphatidylinositol (3,4,5)P_3_ (PIP_3_) in vivo (Figure 1). They form a heterodimeric complex with non-catalytic subunits. Based on the ability of p110 to bind to the type of non-catalytic adapter, they are further distinguished as class IA members (p110α,β,δ) which are associated with p85 type adapters and class IB p110γ which is found in complex with p101 or p87 (also known as p84). Due to the type of adapter, class IA enzymes have been recognized as receptor tyrosine kinase (RTK)-regulated effectors whereas the class IB PI3Kγ is considered to be selectively controlled by G-protein-coupled receptors (GPCRs). However, GPCRs also control the class IA PI3Kβ. In fact, GPCRs regulate the two different PI3Ks via interaction between G-protein βγ dimers and the catalytic p110 isoforms of PI3 kinases β and γ thereby determining a variety of isoform-specific critical roles within the large universe of GPCR-regulated cellular responses. Whereas the specific functions of PI3Kβ were recently reviewed [4], we will focus first on the complex regulation of PI3Kγ by GPCRs and small GTPases, and its non-canonical scaffold functions. We then summarize the biology of PI3Kγ signaling in distinct tissues and in human diseases. Particular attention is given to the role of the non-catalytic subunits p101 and p87. The evidence to consider these adaptors as important elements in assigning different functions to the PI3Kγ variants is discussed.

## 2. Identification of PI3Kγ and Its Tissue Distribution

PI 3-kinases were originally discovered as phosphatidylinositol-specific lipid kinases under the control of tyrosine kinases [5]. However, by the late 1980s, evidence accumulated showing that PI3K activity was regulated by GPCRs in various cellular systems such as neutrophils or platelets [6,7,8]. Two distinct Gβγ-activated PI3K activities were identified, a p85-associated entity that was synergistically stimulated by GPCRs and RTKs, and a distinct RTK-insensitive pool devoid of p85 but still responsive to Gβγ [9,10,11,12]. While the former species was later identified as PI3Kβ the latter was characterized as PI3Kγ [13,14].

### 2.1. PI3Kγ—Catalytic and Regulatory Subunits

What was initially termed PI3Kγ turned out to be only the catalytic subunit p110**γ** (official symbol: PIK3CG; gene located on human chromosome 7; 1102 amino acids (human)), which was cloned from a human U937 complementary DNA (cDNA) library [15]. This monomeric PI3Kγ (p110γ) exhibited hallmarks of a class I PIK catalytic subunit such as specific phosphorylation of various phosphoinositides at the D-3 position of the inositol ring and enzymatic inhibition by nanomolar concentrations of wortmannin [16,17]. However, it failed to bind p85 adapter molecules, and was therefore reminiscent of a partially purified PI3-kinase from myeloid-derived cells [11]. Initial reports suggested the presence of a Pleckstrin homology (PH) domain and regulation by both GTP-activated Gα and Gβγ dimers of heterotrimeric G proteins [18,19]. This pattern of regulation was similar to that of phospholipase C-β (PLC-β), which led to the designation of PI3Kγ as a class IB PI3K. However, neither the PH-domain [20] nor activation by Gα isoforms was confirmed [13,21,22,23].

Purified recombinant expressed p110γ was stimulated by Gβγ complexes at EC_50_ concentrations in a low nanomolar range, similar to those required for regulation of other Gβγ effectors [23]. Moreover, p110γ was able to functionally link GPCR to intracellular effector systems such as the Akt (PKB)-, Jun-, or MAPK-signaling pathways in COS-7 cells [24,25,26,27]. However, overexpressed p110γ produced only moderate effects, and other studies reported the inability of monomeric p110γ to convey receptor-induced signaling in cells [21,28,29].

Purification of native PI3Kγ from neutrophils and platelets clarified the situation, which revealed a heterodimeric 220 kDa protein [12,13]. Peptide sequencing and molecular cloning identified the previously reported enzymatic active p110γ as well as a non-catalytic subunit, termed **p101** (official symbol: PIK3R5; gene located on human chromosome 17; 880 amino acids (human)), which showed no obvious similarity to other proteins or known domains [13]. The p101 subunit is thought to be the crucial adapter for Gβγ stimulation of p110γ [13,23,30,31]. The p101–p110γ heterodimer was strongly stimulated by LPA receptors in cells, and by purified Gβγ in vitro [13]. The binding affinity of p101–p110γ for Gβγ was five-fold greater than for p110γ alone, suggesting that p101 acts as PI3Kγ adaptor which sensitizes the enzyme for Gβγ [13].

When overexpressed in mammalian cells monomeric p101 predominantly localizes to the nucleus consistent with a nuclear localization signal (NLS) motif at positions 499–502 (porcine protein) [21,32]; this localization is abolished upon co-expression with p110γ. However, p101 is unstable as a monomer in insect and mammalian cells [21,32,33,34], and the expression level of p101 appears to be dependent on the amount of p110γ. The p101 and p110γ subunits form highly stable heterodimers upon co-expression or reconstitute from individually purified proteins, and are unlikely to dissociate under physiological conditions [35].

The second p110γ regulatory subunit, **p87** (also known as p84; official symbol: PIK3R6; gene also located on human chromosome 17 close to PIK3R5; 754 amino acids (human)) was identified by genetic approaches based on homology to p101 [32,34,36]. It shows an overall sequence similarity of 24% and up to 37% mainly in the N- and C-terminal regions. Unlike p101, purified p87 is stable when expressed in insect or mammalian cells, and forms a tight but reversible association with p110γ [34]. Although p87 like p101 is not structurally characterized, mutagenesis studies suggest that it interacts with both the helical and N-terminal domains of p110γ [33,35,37]. Unlike the highly stable p101–p110γ dimers, p87 and p110γ undergo reversible dimerization. The p87–p110γ complex partially dissociates upon immunoprecipitation with anti-p110γ antibodies, and dissociation of the p87–p110γ complex is caused by increasing concentrations of free p101, leading to subunit exchange [33]. This p87–p110γ dissociation was also observed in mast cells, where protein kinase Cβ (PKCβ)-mediated phosphorylation of S502 in the helical domain of p110γ reduces the affinity between p110γ and p87 [37].

### 2.2. Tissue Distribution of PI3Kγ

In contrast to the class IA G-protein-sensitive p110β isoform which is ubiquitously expressed, although it seems to be absent or present at low levels in some cell types, such as B- and T-lymphocytes [38] the p110γ expression was originally thought to be largely restricted to immune cells of hematopoietic origin. Now it is considered to be widely distributed throughout the organism including the heart and the central nervous system [14,15,33,39,40,41,42,43,44,45]. The underlying data should be taken with some caution because of the potential contamination of the tissues with blood cells known to show high expression levels of PI3Kγ. Therefore, the detection of the enzyme in specific cells such as endothelial cells, microglia, neurons, tubular cells of the kidney, and exocrine and endocrine pancreatic or prostate gland cells seems to be more reliable [15,42,43,46,47,48,49]. Interestingly, PI3Kγ expression is deregulated in a variety of solid tumors, where it contributes to tumorigenesis and invasion [50,51,52]. The p87 subunit is highly expressed in the immune system but also in other tissues and organs, including the heart and central nervous system (Table 1) [28,33,34,36]. While its mRNA and protein expression level in bone marrow-derived mast cells (BMMC) depend on the presence of p110γ, this is not seen in other bone marrow-derived cells [28]. It remains to be clarified whether monomeric PI3Kγ subunits occur in cells as was observed for the p85 adapter of the class IA enzymes [53]. The p101 is also found in high concentration in the immune system, but was below the detection limit in other body compartments suggesting a more restricted expression profile (see Table 1; see below). Because p101 expression levels increased upon stimulation whereas p87 levels remained unchanged (see below), it was speculated that the p87 variant of PI3Kγ represents a constitutively expressed enzyme, whereas the PI3Kγp101 variant serves as an inducible expressed counterpart [32].

## 3. Regulation of PI3Kγ by Gβγ

### 3.1. Regulation of PI3Kγ by Heterotrimeric G-Proteins

**Figure 2:**
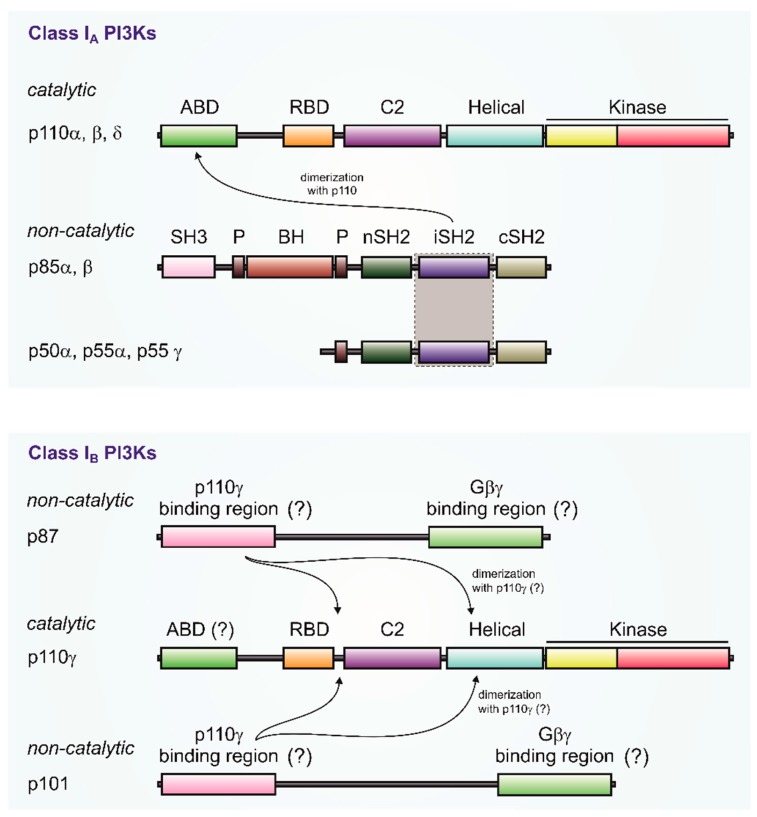
Modular organization of class I PI3K subunits. Class I PI3Ks are heterodimeric lipid kinases consisting of catalytic and non-catalytic subunits. Class I PI3Ks are further subdivided into class IA and class IB. Catalytic subunits of class IA (p110α, p110β or p110δ) form heterodimeric complexes with any of the p85-related non-catalytic subunits (p50α, p55α, p55γ, p85α, or p85β). Class IA p110 subunits comprise an adaptor-binding domain (ABD), a Ras-binding domain (RBD), a C2 domain, a helical domain, and a kinase domain which is subdivided into N-terminal and C-terminal lobes. All p85-related subunits contain N- and C-terminal Src homology 2 domains (nSH2 and cSH2) separated by a coiled-coiled inter-SH2 domain (iSH2) which is responsible for dimerization with p110 ABD. The p85α and p85β subunits additionally possess Src homology 3 domain (SH3) and a Bar cluster region homology domain (BH) which is flanked by two proline-rich regions (P). Class IB p110γ subunits bind non-catalytic p87 or p101 subunits, forming two distinct heterodimeric enzymes. The modular structure of p110γ is similar with class IA p110 subunits. The presence and the role of the ABD is not fully understood. N- and C-terminal regions of p87 and p101 show a high degree of amino-acid similarity and are involved in direct interaction with p110γ and Gβγ, respectively. HDX-MS comparison of heterodimeric PI3Kγ enzymes proposed a role of the RBD-C2 linker and the helical domain in direct interaction with p87 or p101 [37,54].

The catalytic subunits of PI3 kinases γ and β, i.e., p110γ and p110β are both stimulated by G-proteins. For p110β, as well as for the p110α and p110δ isoforms, binding to the regulatory subunit (p85) is mediated by the N-terminal adapter-binding domain (ABD) (Figure 2). The p85 subunit does not bind to G-proteins. The PI3Kγ catalytic subunit, p110γ, can couple to two distinct regulatory subunits, p101 and p87 (Figure 2) [13,34,36]. Whereas p101 exhibits a high affinity toward G-protein βγ dimers, its p87 counterpart show little binding towards Gβγ [29]. The ABD region of PI3Kγ is not structurally characterized, but both the N- and C-terminal regions of p101 and p87 to the Ras-binding domain (RBD) C2 domain linker and the helical domain of p110γ are implicated in binding to its regulatory subunits [32,37,54] (see Figure 2).

#### 3.1.1. Identification of Gβγ-Binding Sites on PI3Kγ

The p110γ and p110β subunits share 35% amino acid identity, with the greatest similarity in the kinase domain [55]. Despite this moderate degree of amino acid homology purified p110γ and p110β exhibit similar sensitivities toward Gβγ-stimulation in the absence of non-catalytic subunits, as indicated by EC_50_ values suggesting analogous binding sites [56]. To get deeper insight into these Gβγ-binding sites in p110γ and p110β site-directed mutagenesis was combined with mapping of contacts between purified proteins by hydrogen deuterium exchange coupled to mass spectrometry (HDX-MS) [54,57]. This approach revealed for both enzymes a flexible loop as the binding site that was not seen in crystal structures. The loop lies in the C2 domain helical domain linker, and their amino acid sequences differ between p110γ and p110β. However, the loops have a pair of basic residues at the C-terminal ends in common which turned out to be important for interactions with Gβγ [54,57]. In the case of PI3Kβ, exchange of these basic residues to Asp eliminates its activation in vitro and in vivo. In accordance, corresponding mutations prevent the activation of p110γ monomers. However, Gβγ was still able to stimulate the p110γ mutant when in complex with p101, although its potency and efficiency was reduced. This may be explained by the observation that the p101 regulatory subunit also binds directly to Gβγ on the one hand and that p101 has its own inherent regulatory functions on p110γ on the other hand (see below) [54,57,58].

#### 3.1.2. Regulation of PI3Kγ Activity by Gβγ

HDX-MS studies also emphasized the lipid environment as an important requisite for Gβγ-induced activation of p101–p110γ and p85–p110β. The presence of lipid vesicles remarkably increased solvent accessibility of the Gβγ-binding loops, indicating that membrane binding exposes the Gβγ site in both enzymes. Contrarily, in the absence of lipids the Gβγ-interacting loop is hidden which is consistent with the finding that antibodies cannot co-immunoprecipitate Gβγ and PI3Kβ in detergent lysates whereas PI3Kβ together with Gβγ can recruit to and co-sediment with lipid vesicles ([4,56] and J. M. Backer and B. Nürnberg, unpublished observations).

Interestingly, in a lipid-free environment the solvent exposure of the Gβγ binding loop of p110γ is decreased upon binding to p101 [54]. This may be interpreted as a protection of the Gβγ binding site in p110γ by p101 when membranes are absent, thereby preventing interactions between p110γ and Gβγ. Conversely, within a lipophilic environment p101 enhances the binding of PI3Kγ to Gβγ through additional contacts to the complex. In line with the role of p101 as a Gβγ adapter p101 mutants missing critical amino-acid residues important for Gβγ binding decreased the potency and efficiency of Gβγ-mediated PI3Kγ activation in vitro. Moreover, when the Gβγ binding sites were mutated in both p101 and p110γ, Gβγ-induced stimulation of PI3Kγ was only minimal. Under in vivo conditions, GPCR ligands were hardly able to stimulate PI3Kγ with mutations of the Gβγ-binding sites in either p110γ or p101 [54,57].

Gβγ binds to both p101 and p110γ [54], but the stoichiometry of the Gβγ PI3Kγ complex is not yet known. In addition, the binding of p110γ to lipid membranes causes a decrease in solvent exposure of a C-terminal helix in the kinase domain, and an increase in solvent exposure of the activation loop [54]. The presence of a C-terminal helix that moves between closed and open conformations is similar to the structure of the class III PI 3-kinase [59]. Surprisingly, membrane binding caused these conformational changes in the absence of Gβγ, and the binding of Gβγ to p110γ in the presence of membranes does not accentuate the changes in the kinase domain C-terminus. These data suggest that activation of p101–p110γ by Gβγ may work in part by enhancing p101–p110γ binding to membranes, thereby increasing the amount of kinase domain that is in the open conformation. However, while constitutive targeting of p110γ to membranes by addition of a C-terminal CAAX motif led to elevated basal activity, the level of activity was less than that caused by Gβγ [21,33,60]. Thus, additional conformational changes in Gβγ-stimulated p110γ must occur. This is perhaps best demonstrated by the observation that p101 binding to p110γ alters the substrate specificity of the enzyme, increasing its utilization of PIP_2_ versus PI as a substrate [31]. Hence, membrane-associated Gβγ stimulates PI3Kγ activity via different mechanisms including membrane recruitment to increase the access to lipid substrates and conformational changes of the lipid kinase to enhance its catalytic activity.

#### 3.1.3. Differential Regulation of p101–p110γ and p87–p110γ by Gβγ

In addition to its contribution to PI3Kγ-Gβγ binding, p101 regulates p110γ activity independently of Gβγ. For example, HEK cells expressing the p110γ-CAAX mutant produced more PIP_3_ upon co-expression with p101, but not with p87 [29], and p101 but not p87 drastically increased enzymatic activity of p110γ upon dimerization under conditions where monomeric and heterodimeric enzymes exhibited equal membrane binding in vitro [33]. Further support for the idea that the Gβγ adapter p101 has inherent regulatory functions comes from studies demonstrating the ability of p101 (i) to enhance Gβγ-induced stimulation of lipid-associated p110γ, (ii) to rescue the stimulatory effect of Gβ_1_ mutants deficient in stimulating p110γ, and (iii) to better protect the inhibitory action of an antibody targeting p110γ than p87 does [33,35,58,61]. These effects were not seen with p87.

**Figure 3:**
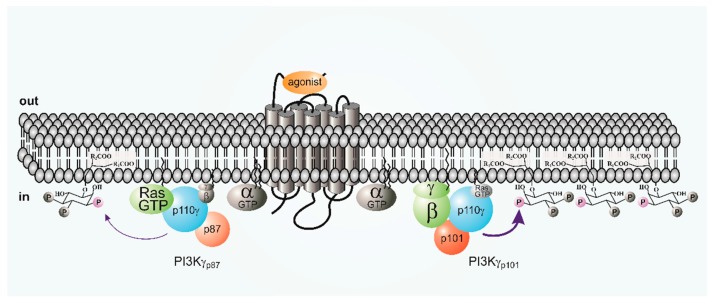
Schematic Regulation of class I PI3Ks. Class IB PI3Kγp87 and PI3Kγp101 are differentially regulated by Ras and by GPCRs via the interaction with Gβγ. Active Ras is indispensable for the translocation of cytosolic PI3Kγp87 to the plasma membrane. In contrast, interaction with Gβγ is sufficient for PI3Kγp101 membrane translocation. Both Gβγ and Ras contribute to the stimulation of the lipid kinase activity of membrane-associated PI3Kγ enzymes.

Initially, p87 was thought to be functionally largely redundant to p101. While it was recognized to be less responsive to stimulation by GPCRs or Gβγ this was explained by a decreased affinity of p87 to Gβγ (Figure 3). [34,36,60]. However, a direct comparison of Gβγ sensitivity of purified preparations of the three PI3Kγ variants showed that p101–p110γ dimers were 20-fold more sensitive to Gβγ than p110γ and p87–p110γ (EC_50_ = 12 versus 214 or 223 nM, respectively). Moreover, p101–p110γ, but not p110γ or p87–p110γ, was efficiently recruited to lipid vesicles by Gβγ [29]. Studies on the role of p87 in the regulation of PI3Kγ by Ras are discussed below.

#### 3.1.4. Gβγ-Isoform Specificity for PI3Kγ Activation

A broad spectrum of G_s_-, G_i_-, and G_q_-coupled receptors are known to control both PI3Kβ and PI3Kγ activity via Gβγ dimers. Regulation of these PI3Ks by G_i_-coupled receptors is best studied, whereas regulation by G_s_- and G_q_-coupled receptors is presumed to produce lower concentrations of free Gβγ [62]. However, it is feasible that any GPCR is capable to stimulate PI3Kγ as long as sufficient amounts of Gβγ complexes are released [63]. Gγ dimers are membrane associated due to the prenylation of Gγ and are, therefore, thought to target PI3Kγ to their substrate in the inner leaflet of the plasma membrane. Consistent with this model, non-prenylated Gβγ dimers still bind to PI3Kγ but do not stimulate enzymatic activity of PI3Kγ in vitro [31,64]. The lipid moiety attached to Gγ may affect the potency of PI3K activation. For example, the C20-isoprenylated Gβ_1_γ_2_ is a much more potent activator of PI3Kγ than retinal transducin Gβγ (Gβ_1_γ_1_), which is also C15-farnesylated [56]. The γ subunits of Gβγ complexes are not only isoprenylated but also methylated at their carboxy-terminal cysteine residues, and PI3Kγ activation was reduced 10-fold for demethylated farnesylated transducin Gβγ, and two-fold for demethylated geranylgeranylated Gβ_1_γ_2_ [65,66].

The regulation of PI3Ks by distinct isoforms of Gβγ was studied for PI3Kγ. Among Gγ subunits, the ubiquitously expressed Gγ_10_ is the least potent. With regard to Gβ, the highly related Gβ_1–4_ isoforms activated PI3Kγ equally well, whereas the more distant Gβ_5_ was inactive [22,56].

### 3.2. Activation of PI3Kγ by Small GTPases

Ras directly binds and stimulates class I PI3Ks due to the presence of a so-called Ras binding domain (RBD) in all class I PI3Ks [67]. The RBDs of p110α, p110δ, and p110γ but not p110β bind to Ras in a GTP-dependent manner [68]. The activation of PI3Kγ by Ras enlarges the range of receptors that can activate this enzyme, including RTKs and Toll-like (TL)/interleukin 1 (IL1) [62,69].

For p110γ, the K_d_ for Ras binding is 2.8 or 3.2 μM for N—or K-Ras-GMPPNP, respectively, and binding of V12-H-Ras increases the activity of p110γ or p110γ–p101 by 23-fold and 20-fold, respectively [70]. Addition of both Gβγ and V12-H-Ras synergistically activates p110γ in the absence or presence of p101. However, in all cases, specific activity is over two-fold greater for p110γ–p101 as opposed to p110γ alone. The crystal structure of Ras-GMPPNP bound to a p110γ mutant with enhanced Ras binding affinity shows conformational changes in the RBD as well as the C2 domain and the C-terminal lobe of the kinase domain. The latter is proposed to increase access of the kinase to the membrane and to change the orientation of helical segments in the phosphoinositide head-group-binding site [70].

In vitro, H-Ras, N-Ras and K-Ras as well as the related R-Ras are able to stimulate PI3Kγ. R-Ras and K-Ras activates PI3Kγ more potently than H-Ras and N-Ras [68,71]. Furthermore, isoprenylation of H-Ras greatly enhances binding and stimulation of PI3Kγ [71,72]. While H-Ras stimulates both p101–p110γ and p87–p110γ the EC_50_ for stimulation of p87–p101 is 50% lower than for p101–p110γ [35]. When expressed in cells, Gβγ stimulation of p87–p110γ but not p101–p110γ is abolished by mutation of the p110γ RBD [29]. It, therefore, appears that Ras acts as an indispensable co-regulator of and “membrane anchor” for the p87–p110γ variant, presumably in response to GPCR-mediated activation of Ras guanine nucleotide exchange factors (GEFs) [62].

The importance of Ras for regulation of PI3Kγ in neutrophils was further emphasized in knock-in mice expressing a p110γ mutant (p110γ^DASAA^; T232D, K251A, K254S, K255A and K256A) that is defective for Ras binding [73,74]. However, it was surprising that the loss of GPCR coupling to PI3Kγ in mice expressing the RBD mutant was more profound than in p101-null mice. It was pointed out that in mammalian cells GPCRs are not generally known to be strong activators of Ras [75]. These data should be interpreted with care, as mutation of five consecutive amino acids in the RBD could affect Gβγ-dependent stimulation of p110γ independently of Ras. Recently, a missense mutation was identified in the Ras-binding domain of p110γ (Val282Ala) in a patient suffering from chronic infections and pelvic pain, which may point to a clinical relevance of Ras p110γ interaction [76].

In addition, Rab8a was reported to interact directly with p110γ through its RBD to modulate Toll-like receptor 4 (TLR4)-driven PI3K and mammalian target of rapamycin (mTOR) signaling in macrophages. This was proposed as a mechanism for biasing the cytokine profile to constrain inflammation in innate immunity [77,78].

All these data illustrate that the p87 and p101 variants of PI3Kγ are stimulated by Ras and Gβγ to different degrees: however, vice versa, these characteristics allow PI3Kγ variants to either discriminate or integrate signals from distinct upstream regulators including RTKs and GPCRs [79]. Moreover, PI3Kγ may be also involved in non-canonical G-protein signaling pathways through GPCR kinase 2 (GRK2)-dependent mechanisms [80,81], and in kinase-independent signal transduction (see below). Finally, p87–p110γ can be activated by the high-affinity imunglobulin E receptor (FcεRI) in mast cells and by RTKs and TLR/IL-Rs in myeloid cells [37,69]. Thus, despite the fact that all class I PI3Ks may produce the same product, combinatorial regulation of these enzymes and in particular the PI3Kγ variants can lead to a remarkable level of signaling specificity [82].

## 4. Protein Kinase Activity of PI3Kγ

All class I PI3 kinases are dual specific enzymes with inherent lipid and protein kinase activities [83,84,85,86,87,88]. A limited number of substrates for PI3K protein kinase enzymatic activity were reported, with autophosphorylation being the best studied [89,90,91]. In case of the class IA members, p110β and p110δ autophosphorylate a serine residue at the C-terminus of the kinase domain (S1070 and S1039, respectively) whereas p110α only phosphorylates S608 in the p85α regulatory subunit. All of these phosphorylation events were shown to down-regulate the lipid kinase activity of these PI3 kinases. A recent report, however, questioned the inhibitory role of S608-p85α phosphorylation [92]. In contrast, autophosphorylation of a serine at the extreme C-terminus p110γ (S1101) seems to have either little or no effect on lipid kinase activity [85,87,93].

One immediate question is whether protein kinase activity of the GPCR-coupled PI3Ks is subject to regulation. For PI3Kβ, the data are reported are inconclusive [89,94]. Neither Gβγ nor phosphotyrosyl peptides lead to enhanced PI3Kβ autophosphorylation [85]. However, the effect of Rac1 and cell division control protein 42 homolog (CDC42) binding to the p110β RBD domain [68] remains to be determined. Nevertheless, p110β was shown to phosphorylate exogenous protein substrates such as an intracellular fragment of the granulocyte-macrophage colony-stimulating factor (GM-CSF)/IL-3 βc receptor [93].

In contrast, Gβγ significantly stimulates **PI3Kγ** protein kinase activity in a concentration-dependent manner even in the presence of Mg^2+^ in vitro [31,33,58,85,95], with the EC_50_ for Gβγ in the same range as for lipid phosphorylation. Concomitantly, IC_50_ values of classical PI3K inhibitors such as wortmannin were also found to be equal for the protein and lipid kinase activities of PI3Kγ [87]. Although basal autophosphorylation is visible in the absence of lipid vesicles, Gβγ-dependent stimulated autophosphorylation requires both the presence of a lipid compartment and isoprenylation of the Gγ-subunit [85]. Whereas Gβγ weakly stimulates the protein kinase activity of the p110γ monomer, addition of p101 suppresses basal autophosphorylation but greatly enhances Gβγ-stimulated autophosphorylation of p110 [31,58]. In contrast, Gβγ-induced p110γ autophosphorylation is much smaller in the presence of the p87 isoform (A. Shymanets and B. Nürnberg, unpublished).

It is not known whether Ras-proteins stimulate the protein kinase activity of PI3Kγ.

Interestingly, p110γ was reported to phosphorylate p101 in a Gβγ-dependent manner even in the absence of lipid vesicles [95]. Although phosphorylation of p101 was confirmed by another group [85], stimulation by Gβγ was not observed even when lipid vesicles were present. A potential concern in these studies is the use of glutathione-S-transferase (GST)-tagged p101; GST-tagged monomeric p110γ showed a much greater sensitivity to Gβγ-induced stimulation of protein kinase activity than its His-tagged counterpart pointing to a significant impact of the type of tag attached to p110γ. Interestingly, N-terminal His-tag-dependent differences in Gβγ regulation of PI3Kγ protein kinase activity were already reported [94].

A small number of reports described exogenous substrates for PI3Kγ protein kinase activity both in vitro and in vivo. For example, it was proposed that PI3Kγ protein kinase activity targets the MAP kinase pathway [24] and regulates β-adrenergic receptor endocytosis by phosphorylating cytoskeletal non-muscle tropomyosin [96]. However, the biological significance of PI3K-dependent protein kinase activity remains to be determined.

## 5. Non-Catalytic Functions of PI3Kγ

A comparison of knockout versus kinase-dead knock-in mice led to important insights into non-catalytic functions for PI3Kγ [97]. In particular, differences in the phenotypes of protein-deficient (KO) versus kinase activity-deficient (KD) mice uncovered a role for this enzyme as molecular scaffolds that orchestrate cellular signaling complexes independent of lipid kinase activity [97,98]. These kinase-independent adapter or scaffolding functions appear highly selective since the absence of a particular PI3K isoform is unlikely to be restored by remaining ones, and for unknown reasons was clearly documented only for the GPCR-dependent PI3-kinases. Kinase-independent functions may act in concert or may be interconnected with the kinase activity, like PI3Kγ in diet-induced obesity and PI3Kβ in insulin signaling or they may act independently from each other, as shown by PI3Kγ in cardiac contractility [99,100,101,102,103].

Kinase-independent scaffold functions were most intensely studied in the heart, where PI3Kγ was shown to control cyclic adenosine monophosphate (cAMP) homeostasis. Unexpectedly, p110γ-deficient mice displayed an increase in basal cAMP levels that paralleled enhanced cardiac contractility [104]. However, when the cardiac phenotypes of p110γ KO and KD mice were compared, it became evident that p110γ KD animals showed normal cAMP levels and no alterations in cardiac contractility. A first step to understand the underlying mechanism was the observation that the cardiac PI3Kγp87 variant binds via its p87 subunit to phosphodiesterase 3B (PDE3B), an enzyme that degrades cAMP. This complex appeared to be crucial for maintaining physiological cAMP levels [34,45]. Later studies identified a ternary complex consisting of the p87–p110γ PI3Kγ, PDE isoforms and the regulatory and catalytic subunits of protein kinase A (PKA) [103,105]. Thus, the p110γ catalytic subunit acts as a PKA anchoring protein (AKAP) via its direct binding to the regulatory subunit of protein kinase A. The ternary PI3Kγ complex brings PKA in close proximity to PDE in order to tightly regulate PKA activity. The final level of complexity is the phosphorylation of p110γ at Thr1024 by PKA, which inhibits its lipid kinase activity.

PI3Kγ has additional kinase-independent functions in the cardiovascular system. For example, p87-PDE3B-signaling complexes were identified in human arterial endothelial cells and described to contribute to a dynamic cAMP-dependent regulation of cell adhesion, spreading, and tubule formation [106]. Post-ischemic neovascularization was linked to PI3Kγ scaffold functions in endothelial progenitor cells [107]. The scaffold functions of PI3Kγ may also be responsible for mediating sepsis-induced myocardial depression during inflammation-induced systemic inflammatory response syndrome [108].

Kinase-independent functions for PI3Kγ were described in other physiological contexts [102]. The kinase-independent link between PI3Kγ and the PDE/PKA axis was proposed to play a role in microglial responses after focal brain ischemia [109,110], and in the regulation of cAMP response element-binding protein (CREB)-mediated transcriptional responses in noradrenergic neurons of the locus coeruleus [47]. Dysregulation of PI3Kγ in this setting provokes an attention-deficit/hyperactivity disorder (ADHD) phenotype in mice, including deficits in the attentive and mnemonic domains, typically hyperactivity and social dysfunctions. In white adipose tissue PI3Kγ is a kinase-independent negative regulator of PKA-dependent hormone sensitive lipase activation [41]. Finally, p110γ regulates integrin α_IIb_β_3_ activation in platelets through a non-catalytic signaling mechanism [111], although in this case the mechanism remains to be addressed.

All these findings emphasize a so-called “double identity” for the GPCR-regulated PI3-kinases, which act not only as classical lipid kinases, but also as adapters or scaffolds orchestrating signaling events independently of enzymatic activity [98]. While upstream activators tightly govern the enzymatic activity of these enzymes, future studies will determine whether their scaffolding functions are also subject to dynamic regulation. For PI3Kγ, it will be particularly interesting to understand whether scaffolding functions are restricted to the p87–p110γ dimer.

## 6. Biological Functions of PI3Kγ

Based on its apparent restriction to the hematopoietic system, early studies on PI3Kγ focused on the immune system. More recent works demonstrated a broader expression profile and important roles in multiple physiological systems, including cardiovascular, endocrine, and neuronal functions as well as roles in malignancy (Table 1). These include both direct functions and indirect effects on the immune system.

### 6.1. Immune System

Although all four isoforms of class I PI3K are found in cells of hematopoietic origin, PI3Kγ and PI3Kδ play critical functions in these cells. PI3Kγ is considered to preferentially regulate the innate immune system, as its expression is highest in the myeloid lineage, whereas PI3Kδ is highly expressed in T- and B-cells and is more important in the adaptive immune system [38,39]. In particular, altered regulation of PI3Kγ in neutrophils, eosinophils, macrophages, mast cells, and dendritic cells is implicated in multiple disease states.

Of the two PI3Kγ regulatory subunits, p101 is more widely expressed in the immune system with highest levels found in bone marrow, lymph nodes, spleen, and thymus [28,33,34]. Accordingly, immune cells such as neutrophils or eosinophils express high levels of p101. In contrast, mast cells are almost devoid of p101 [28,37]. Interestingly, reports suggest a temporal diverse expression of the regulatory subunits with an induced upregulation of p101 in immune cells and heart tissues while p87 protein levels remained unchanged [33,112]. Most recently, it was demonstrated that macrophage migration inhibitory factor (MIF) up-regulates the expression of p101 in THP-1 monocytes and HL60 neutrophil-like cells [113]. Hence, it is tempting to consider p101 as part of an inducible PI3Kγ but further work needs to explore this possibility.

**Neutrophils** were intensively studied with regard to signaling functions of PI3Kγ. Two critical PI3Kγ-dependent functions in neutrophils, chemotaxis and reactive oxygen species (ROS) production are regulated by distinct isoforms of PI3Kγ [114,115,116]. Loss of p110γ, p101 or p87 in neutrophils obtained from the corresponding KO mice led to reductions in GPCR-stimulated PIP_3_ production and Akt activation. However, GPCR-regulated chemotactic responses were specifically reduced in neutrophils lacking p101, but not p87 (Table 2) [73,74]. Stimulation of neutrophils with the GPCR ligand *N*-formylmethionyl-leucyl-phenyalanine (fMLP) leads to PI3Kγ-dependent accumulation of the primary lipid products PIP_3_ and PI4,5P_2_ at the leading edge of migrating cells [117]. This coupling of PIP_3_ production to motility requires the spatially restricted activation of PI3Kγ, since expression of an isoprenylated mutant of p110γ resulted in a constitutive but non-polarized membrane association and significant impairment of directional cell migration in response to chemoattractants [118]. These data show that localized activation of PI3Kγ at the leading edge, through Gβγ interactions with p101 and p110γ, is required for maximal neutrophil chemotaxis. Neutrophil recruitment to blood vessel walls in a lipopolysaccharide (LPS)-induced model of acute lung injury model also required the expression of PI3Kγ in endothelial cells [119], suggesting both immune cells and stromal cells contribute to this process. 

While chemotaxis promotes the recruitment of neutrophils to sites of infection, ROS production is a key component for the bactericidal response of neutrophils to pathogens. ROS production was specifically reduced in neutrophils lacking p87 (Table 2). [73,74]. Given that p87 was implicated in Ras activation of PI3Kγ [29], and that mutation of the RBD of p110γ leads to a loss of GPCR-stimulated ROS production [74], it seems likely that Ras activation of the p87–p101 PI3Kγ is the major driver of ROS release.

**Eosinophils.** Eosinophilic inflammation accompanied by elevated immunoglobulin E (IgE) levels, and bronchial hyper reponsiveness are hallmarks of allergic asthma. Several studies demonstrated an impact of PI3Kγ-deficiency or pharmacological PI3Kγ inhibition on eosinophilic chemotaxis and/or maintenance of eosinophilic inflammation in vivo [121,122,123,124,125]. In contrast, reports from ovalbumin-sensitized murine models of methacholine-induced asthma were discordant: mice lacking PI3Kγ exhibited a significant reduction of bronchial hyper responsiveness in some studies but not in others [126,127,128]. While PI3Kδ also seems to mediate eosinophil migration and to contribute to the regulation of allergen-specific IgE production, deficiency of both PI3Kδ and PI3Kγ showed a massive effect indicating their cooperation [127]. However, the PI3Kγ/δ KO mice also displayed marked eosinophilic inflammation, suggesting that dual inhibition of PI3Kγ and PI3Kδ might result in inappropriate unwanted side effects [121].

**Macrophages** act in concert with neutrophils at the front line of innate immunity, and interact with components of the acquired immune system. PI3Kγ was found to be involved in various steps of macrophage activation and function [39,114,129]. This includes FcRγ signaling, chemotaxis, release of ROS, secretion of inflammatory mediators, and phagocytosis of pathogens [130,131,132,133,134]. In addition, recent studies show that in tumor-associated macrophages (TAMs), inhibition of PI3Kγ blocks the immunosuppressive effects of TAMs, enhancing the activation of cluster of differentiation 8 (CD8)^+^ T-cells and responses to immune checkpoint inhibitors [135,136,137]. Loss of PI3Kγ expression also blocks macrophage-mediated tumor metastasis in a number of tumor models [138,139]. The effects of PI3Kγ inhibitors on anti-tumor immunity are similar to effects seen with inhibitors of PI3Kδ [140]. These studies may open the possibility to reduce tumor growth and to improve survival of patients when current immunotherapies are combined with novel PI3Kγ and/or PI3Kδ inhibitors.

**Mast cells** are tissue-resident cells involved in host defense and tissue repair under physiological conditions, but whose inappropriate activation leads to type I allergic conditions such as airway inflammation, eczema, and anaphylaxis. A key feature of mast cells is their high content of granules containing histamine and heparin, which are released upon stimulation of Fcε receptors by IgE in an exocytic process termed degranulation. IgE-stimulated mast cell degranulation is synergistically enhanced by adenosine, which stimulates the G_i_-coupled A_3_ adenosine receptor via a PI3Kγ-dependent autocrine activation loop [141]. Interestingly, since mast cells do not express the p101 isoform, PI3Kγ-dependent degranulation is transmitted by the p87-containing variant. Surprisingly, whereas reconstitution of PI3Kγ-null mast cells (which show loss of both catalytic and regulatory subunits) with p110γ and either p87 or p101 could reconstitute Akt activation and cell migration, degranulation was only restored in cells expressing p87 [28]. Expression of p110γ with either p87 or p101 lead to accumulation of PIP_3_ in the plasma membrane, but PIP_3_ production in the p87–p110γ cells was dependent on lipid rafts, and led to the endocytosis of PIP_3_ to intracellular vesicles. PI3Kγ-deficient mast cells display defective chemotaxis which can be rescued by expression of exogenous p110γ together with the p87 variant suggesting some overlapping functions between the non-catalytic PI3Kγ subunits [40]. These data suggest that p87 targets PI3Kγ to specific regions of the plasma membrane, leading to internalization of PIP_3_ and degranulation.

**Dendritic Cells**. Although both PI3Kγ regulatory subunits are expressed in dendritic cells (DCs), most studies focused on the catalytic subunit whereas specific roles for p87–p110γ and p101–p110γ were not described in these cells [34]. PI3Kγ is an essential intrinsic regulator of conventional development of DCs in the lung and immune organs, but not other tissues. In this pathway, the Fms-like tyrosine kinase 3 ligand receptor (FLT3) together with Ras activated PI3Kγ controls DC development in immune organs and the lung, but not in other non-immune tissues [142]; in immune organs, PI3Kδ may also be involved. PI3Kγ is required for DC responses to chemokines or oxidative stress, and for the migration of antigen-loaded DCs from the periphery to draining lymph nodes in contact- and delayed type-hyper sensitivity reactions [143]. Migration of bone marrow-derived murine DCs treated with hydrogen peroxide also depends on PI3Kγ [144]. This role for PI3Kγ in DC migration is apparent in an experimental murine model of autoimmune encephalomyelitis promotion, where the absence of PI3Kγ in DCs led to defects in migration with a failure of full T cell activation following T-cell antigen receptor (TCR) ligation [145,146]. Recently, it was shown that PI3Kγ-defective lung DCs lead to deficient CD8^+^ T-cell priming, which the authors linked to a delayed viral clearance and increased morbidity during respiratory infections with influenza virus [147].

**T- and B-cells**. Despite the dominant role played by PI3Kδ in thymocytes, mice deficient for PI3Kγ show an impaired thymocyte development, as well as reduced T-cell proliferation and cytokine production [116]. The reduced cytokine production is a result of impaired T-cell receptor signaling [148]. Furthermore, roles for PI3Kγ in T- and B-cells also became apparent when PI3Kδ was absent or inactive, indicating overlapping roles of the two PI3-kinases [130,149,150,151]. This is of particular interest for developing dually acting drugs on both kinases to treat hematological malignancies [152].

### 6.2. Platelets

Physiological activation of platelets results in a marked production of PIP_3_, which is important for various platelet functions including the formation of stable thrombi [153]. Whereas PI3Kβ was identified to be particularly responsible for PIP_3_-dependent thrombus growth and stability, the impact of PI3Kγ seems rather limited [154,155]. In platelets, both PI3K isoforms act downstream of the G_i_-coupled P2Y_12_-receptor, with PI3Kβ being the dominant isoform. In contrast to P2Y_12_ receptor- and Gα_i2_-knockouts, animals deficient for PI3Kγ had no bleeding phenotype but were protected from acute pulmonary thromboembolism caused by infusion of ADP [156,157,158]. PI3Kγ seems to cooperate with PI3Kβ for G_i_-stimulated integrin activation through its catalytic activity [111]. In addition, α_IIb_β_3_-activation was inhibited by loss of PI3Kγ expression but not by treatment with PI3Kγ inhibitors, suggesting a kinase-independent mode of action [111].

### 6.3. Cardiovascular System

PIP_3_ is an important second messenger in heart function; thus, various class I PI3K isoforms play roles in cardiomyocyte function. PI3Kα has a central role in regulating cardiac postnatal growth and survival, promoting physiological hypertrophy and sustaining systolic functions in adults but also protecting the heart against pathological remodeling and failure [101]. Together with PI3Kβ it maintains the organized network of T-tubules that is vital for efficient Ca^2+^-induced calcium release and ventricular contraction [159].

In contrast to the class IA PI3-kinases, cardiac PI3Kγ negatively regulates inotropic responses downstream from the β-adrenergic signaling cascade via kinase-dependent and -independent mechanisms. In cardiomyocytes, both the protein and lipid kinase activities of PI3Kγ are required to shut off agonist-dependent β-adrenergic receptor (β-AR) signaling through (i) inhibition of protein phosphatase 2A activity, which promotes receptor desensitization and internalization, (ii) cooperation with GPCR kinase-2 (GRP-2, also known as β-adrenergic receptor kinase-1 (β-ARK-1)), which induces receptor desensitization, and (iii) phosphorylation of non-muscle tropomyosin, enabling β-AR internalization [96,112,160,161]. Thus, increases in PI3Kγ lipid kinase activity correlate with the downregulation of cell surface β-AR receptors and the uncoupling of receptors from downstream signaling; these changes are hallmarks of heart failure. The increase in PI3Kγ activity is due both to a significant upregulation of PI3Kγ expression levels as well as a decreased inhibition of its lipid kinase activity via protein kinase A (PKA)-mediated phosphorylation [112], which along with PDE binds directly to PI3Kγ (discussed above). The PI3Kγ-PKA-PDE complex also operates in a negative feedback loop, as PDE antagonizes β-AR activation of PKA [100,105]. Thus, along with studies showing that adoptive transfer of PI3Kγ KO bone marrow limits fibrosis and left-ventricular dilatation after aortic constriction [100], these studies point to PI3Kγ as a potential drug target in the treatment of heart failure in the context of pressure overload or diabetes [162,163,164,165].

The role of PI3Kγ in the preservation of cardiac function following ischemia reperfusion is complex, as the enzyme appears to play both positive and negative roles in the response to ischemic damage. Post-ischemic pharmacological inhibition of PI3Kγ together with PI3Kδ limits ischemia/reperfusion injury through their functions in various cell types including cardiomyocytes and immune cells [166]. PI3Kγ contributes to myocardial preconditioning, an experimental model in which brief exposure to ischemia or adenosine is protective against the effects of long-term ischemia [167]. In contrast, loss of PI3Kγ enzymatic activity or PI3Kγ expression inhibits reparative neovascularization, with increased infarct size and reduced left-ventricular function, in a mouse model of myocardial infarction [168]. Haubner et al. showed that reconstitution of mice with bone marrow from PI3Kγ KO, but not PI3Kγ KD, mice led to increased infarct size in an ischemia/reperfusion model [169]; the authors questioned the utility of PI3Kγ inhibitors in the treatment of ischemic cardiac disease. Interestingly, a p110γ-specific inhibitor did reduce necrosis during renal ischemia/reperfusion injury in the kidney, suggesting that PI3Kγ plays many tissue-specific roles in the response to ischemia [170].

Inhibition of PI3Kγ either pharmacologically or genetically is beneficial in mouse models of vascular injury and atherosclerosis. PI3Kγ inhibitors reduced early and advanced atherosclerotic lesions in Apolipoprotein E-deficient and low-density lipoprotein-deficient mice, respectively [171]. Similarly, adoptive transfer of PI3Kγ KD CD4^+^ T-cells into Rag2-mice reduced vascular occlusion and an associated T-helper cell 1 (Th1) immune response in a mouse model of intimal hyperplasia [172]. In contrast, endothelial regeneration and vascular repair in the lung after sepsis-induced inflammation were reduced in PI3Kγ KO mice [173].

The four class I PI3K kinases were shown to selectively link RTKs and GPCRs to stimulation of L-type calcium channels in the vasculature [174,175]. Under in vitro conditions, angiotensin II stimulated L-type calcium channels in vascular myocytes through AT_1A_ receptors, Gβγ and PI3Kγ [176,177]. Mechanistically this is accomplished through the translocation of intracellular calcium channels to the plasma membrane, an intracellular trafficking process that specifically requires the β_2_ subunit of L-type calcium channels [177]. Correspondingly, mice devoid of PI3Kγ were protected from angiotensin II-induced hypertension [178]. Moreover, the relevance of PI3Kγ for regulating blood pressure was further demonstrated in normotensive and hypertensive mice using small- molecular inhibitors of PI3Kγ [179]. Recently, relaxin-2, a structurally insulin-related peptide, was shown to exhibit vasodilatory effects on murine mesenteric arteries through a Gα_i2_-controlled PI3Kβ-and PI3Kγ-dependent pathway via production of nitric oxide (NO) [180]. Relaxins are implicated in different aspects of the cardio-metabolic syndrome. Interestingly, screening of 2.500 individuals at risk for type-2 diabetes revealed genetic variations in p110γ that correlated with high-density lipoprotein (HDL)-cholesterol, suggesting a role for PI3Kγ in atherogenesis [181].

Taken together, these studies suggest that PI3Kγ may be a promising target to fight cardiovascular diseases such as heart failure, hypertension, atherosclerosis, or diabetic cardiomyopathy, among others. However, given the complex and multifaceted roles of PI3Kγ in these diseases, with roles in both physiological and pathophysiological responses, it remains a tremendous challenge to beneficially intervene in a specific PI3Kγ pathway without causing unwanted PI3Kγ-dependent side effects at a different site.

### 6.4. Metabolism

PI3Kγ is implicated in the control of metabolic functions at multiple sites. In particular it contributes to the control of glucose and lipid homeostasis [99]. In pancreatic β-cells PI3Kγ facilitates insulin secretion. Mechanistically, it is assumed to enhance insulin secretion via limiting the cortical actin barrier that prevents exocytosis of insulin granules [49,182,183]. These findings suggest an antidiabetic role for PI3Kγ that apparently contradicts reports demonstrating pro-diabetic effects in diet-induced obesity and thermogenesis, hepatic steatosis, metabolic inflammation, and insulin resistance [41,184]. In addition, PI3Kγ deficiency reduces pancreatic β-cell apoptosis [185]. Given the role of PI3Kγ in inflammation, and the role of inflammation in obesity-induced insulin resistance [186], it is well understandable that PI3Kγ affect insulin signaling through the immune system [185]. However, the two groups reached opposite conclusions as to this point; adoptive transfer experiments with wild-type or PI3Kγ KO bone marrow suggested that the lean phenotype was due to PI3Kγ signaling in non-hematopoietic cells [41], whereas bone marrow-specific PI3Kγ KO suggested a key role for PI3Kγ in hematopoietic cells [184]. An additional kinase-independent role for PI3Kγ in the action of hormone-sensitive lipase in adipocytes was also described [41]. Finally, both PI3Kγ and PI3Kβ were found to act centrally by inhibiting melanocortin 4 receptor signaling. Central inhibition of PI3Kγ and PI3Kβ lad to increased sympathetic nervous system signaling to white adipose tissue, resulting in increased lipolysis and browning, and increased energy expenditure and weight loss [187]. Little is known about the impact of the non-catalytic PI3Kγ subunits in diabetes mellitus (DM). Recently, p87 was reported to be one of the key genes in murine liver tissue associated with the development of type II DM [188], whereas computational analysis suggest that the human p101 belong to a group of highly disordered proteins related to type II DM [189]; however, nothing is known about the relevance of these findings.

### 6.5. Nervous System

Although PI3Kγ is considered to be an enzyme confined to hematopoietic expression emerging evidence suggests distinct roles in the nervous system. This was not only demonstrated for central nervous system (CNS)-dependent control of weight (see above) but also for various sensory functions. In a dry skin model of itch, PI3Kγ inhibition reversed scratching behavior. It was, therefore, speculated that GPCRs are expressed by the central terminals of dorsal root ganglia (DRG) nociceptive afferents and transmit itch via PI3Kγ [190]. Using a carrageenan-induced inflammatory pain model induced in rat hind paws together with isoform-specific inhibitors, it was shown that a PI3Kγ-blocker selectively administered before treatment reduced the carrageenan-induced pain behavior and spinal expression of spinal c-Fos as indicator of nociception [191]. Conversely, anti-nociceptive effects triggered by morphine-induced activation of peripheral opioid receptors in primary nociceptive neurons of mice were found to be initiated by PI3Kγ and its effector protein kinase B (PKB) [46]. As a result, neuronal nitric oxide synthase (nNOS) is activated, thereby producing NO, which in turn induces an increase in K_ATP_ channel currents that causes hyperpolarization of nociceptive neurons.

Interestingly, PI3Kγ was predicted to be critical for *N*-methyl-d-aspartate (NMDA) receptor-dependent long-term depression and some forms of cognitive function suggesting roles in synaptic plasticity and mediating behavioral flexibility [170]. Moreover, based on mouse data PI3Kγ was assigned an essential role in attention-deficit/hyperactivity disorder (ADHD) [47]. In particular, lack of PI3Kγ displayed deficits in attentive domain and mice were hyperactive, together with an unbalanced catecholaminergic activity in the fronto-striatal areas, receiving projections from the locus coeruleus (LC). It was speculated that LC function is regulated by PI3Kγ through a kinase-independent mechanism that affects the cAMP pathway including PDE4B and the transcription factor CREB [192].

### 6.6. Cancer

Class IA PI3-kinases and in particular, PI3Kα mutants are considered as driving forces of tumor growth whereas the PI3Kγ isoform is a less established candidate. By contrast to p110α and similar to p110β overexpression of p110γ transform cells in vitro, this process still needs upstream signaling input since mutating the Gβγ-binding site on p110γ or p101 severely affects the cell transforming efficiency [54,193]. Nonetheless, copy number gain or increased expression levels rather than mutations were associated with various patients’ tumors such as leukemia and medulloblastoma, as well as breast, liver, pancreatic, ovarian, clear-cell renal carcinoma or prostate cancer [52,152,194,195,196,197]. It is worth mentioning that recurrent mutations of the *PIK3CG* gene were reported to occur in a considerable number of patients suffering from biliary cancer, metastatic prostate cancer or renal cell carcinoma although the functional consequences remain to be established [50,198,199,200]. Aside from a deregulated function in tumor cells PI3Kγ contributes in multiple ways to tumor growth and metastasis. Some of these augmentable PI3Kγ effects may be therapeutically exploited by PI3Kγ-targeted immuno- (as already outlined, see above) or angiogenic inhibition or by targeting the effect of PI3Kγ on the fibrous stromal tissue around tumors to fight malignancies [138,201,202]. Lack of PI3Kγ in mice indicated specific roles in endothelial cells, as endothelial progenitor cells displayed reduced integration into endothelial networks required for proper capillary formation [50]. Furthermore, blocking PI3Kγ resulted in anti-angiogenic effects in cancer due to an inhibition of tumor-associated myeloid cells [201]. This is of particular relevance since resistance to vascular endothelial growth factor (VEGF)-targeted therapy in tumors is partially mediated by these myeloid cells in a PI3Kγ-dependent manner. In spite of the multi-pronged strategies the first PI3Kγ inhibitor still awaits development to reach the patient (see also above) [38,203]. 

Of some pathophysiological relevance appear observations that overexpression of the non-catalytic p101 subunit of PI3Kγ led to oncogenic cellular transformation and malignancy, whereas the loss of p101 was reported to be sufficient to reduce in vivo tumor growth and metastasis to a similar extent to that of p110γ [51,204]. Of note upregulated p101 expression was associated with the progression of ovarian cancer chemoresistance in patient-derived xenograft murine models of ovarian cancer [205]. Interestingly, in contrast to the proposed tumor-promoting potential of p101 and p110γ, the Thr-607-phosphorylated p87 adapter may harbor tumor suppressor activity and attenuation of cell migration by forming a negative regulatory complex with p110γ to control PI3Kγ signaling [51,206]. Moreover, agonist-induced nuclear localization of PI3Kγ was reported, indicating an unexplored regulatory role for PI3Kγ in this compartment [207].

## 7. Pharmacological Inhibitors of PI3Kγ

PI3-kinases play crucial roles in many pathophysiological conditions such as cancer, and inflammatory or autoimmune diseases. It is therefore not surprising that in particular class IA PI3- kinases are considered to be attractive pharmacological targets to treat these pathological disorders, resulting in a remarkable repertoire of small-molecule inhibitors [3,207,208,209,210,211,212]. Furthermore, studies demonstrating profound effects of PI3Kγ knock outs on murine inflammatory disease models led to great interest in the immunological functions of PI3Kγ and the use of PI3Kγ inhibitors to treat inflammatory disorders [38,114,115,116,213]. Decades later, the number of drugs reaching the clinic is sobering. The development of many of these compounds was not pursued due to insufficient efficacy on the one hand and serious unwanted drug effects on the other hand [2,214]. Underlying mechanisms of the failure of highly selective PI3K inhibitors include the development of resistance due to mutations and/or bypassing by parallel compensatory pathways, among others [2]. In contrast, strategies using pan-PI3K inhibitors face the problem of severe unwanted drug effects in patients, resulting in a negative risk/benefit evaluation [2,3,214]. One current approach to circumvent these disadvantages is the development of new generations of highly specific PI3K inhibitors selectively targeting a particular PI3K isoforms as part of a combination therapy to prevent compensatory pathways. For PI3Kγ new classes of specific inhibitors are being generated [212,215,216]. They are designed to block the enzymatic activity of the enzyme. However, this approach may not discriminate between the two PI3Kγ variants which share the same enzymatic p110γ subunit but have different regulatory subunits and are, hence, hypothesized to exhibit separate cellular function (see above). A more profound understanding of the function, regulation, and biological role of PI3Kγ variants by their regulatory subunits p101 and p87, as well as the impact of the non-enzymatic functions of PI3Kγ in signal transduction will foster new concepts for intervening in enzymatic and non-enzymatic PI3Kγ signaling [35]. This may include drugs which selectively act on the regulatory p101 subunit or the interface between the p87 and p110γ subunit rather than the catalytic site of the p110γ subunit allowing an advanced level of isoform specificity among the class IB PI3Kγ variants.

## Figures and Tables

**Table 1:** Transcript distribution of PI3Kγ subunits p87 and p101 in human tissue and organs expressing p110γ (data taken from Reference [33]). The ratio of p87 or p101 mRNA to GAPDH mRNA is depicted: not detected (n.d.) <0.001; + 0.001 to 0.005; ++ 0.005 to 0.01; +++ >0.01.

Organ/Tissue/Cell Type	p87	p101
***Immune System***		
Bone Marrow	+++	+++
Lymph Node	+++	+++
Plasma Blood Leukocytes	+++	+++
Spleen	+++	+++
Thymus	++	+++
Tonsil	++	+
***Cardiovascular System***		
Heart	+	n.d.
Intracranial Artery	+	n.d.
Vena Cava	+	n.d.
***Metabolic Organs***		
Colon	+	+
Duodenum (descending part)	+	+
Esophagus	+	n.d.
Fat	++	+
Intestine (small)	++	+
Liver	n.d.	+
Pancreas	+	n.d.
Rectum	+++	n.d.
Stomach	++	++
***Nervous System***		
Brain	+	+
Optic Nerve	+	n.d.
Pituitary Gland	+	+
Retina	+	+
Spinal Cord	+	+
***Others***		
Lung	++	+++
Mammary Gland	+	n.d.
Ovary	+	n.d.
Prostate	++	+
Skin	++	+
Urethra	++	n.d.

**Table 2:** Phenotype of p87and p101 knockout in mice [73,120].

	p87^−/−^	p101^−/−^
Thymocytes	not impaired	requirement for β-selection during thymocyte development
Neutrophils	impaired PIP_3_ production and Akt phosphorylation, as well as less ROS formation, but normal migration	impaired PIP_3_ production, as well as Akt phosphorylation and migration, but normal ROS formation
